# Association of Subjective Oral Status and Oral Health Behaviours With a History of Systemic Disease Among Community-Dwelling Older Adults

**DOI:** 10.3290/j.ohpd.c_2807

**Published:** 2026-09-04

**Authors:** Rumi Nishimura, Haruka Fukutani, Marin Ishigami, Maho Omoda, Mayuka Asaeda, Yukoh Muraki, Noriyasu Uchibori, Mariko Naito

**Affiliations:** a Rumi Nishimura Assistant Professor, Department of Oral Epidemiology, Graduate School of Biomedical and Health Sciences, Hiroshima University, Hiroshima, Japan. Discussed the results, reviewed and edited the manuscript.; b Haruka Fukutani Dental Hygienist, Fujisawa City Health Center, Kanagawa, Japan. Conceived and designed the study, analysed the data, and wrote the manuscript.; c Marin Ishigami Dental Hygienist, Minato Public Health Center, Aichi, Japan. Discussed the results, reviewed and edited the manuscript.; d Maho Omoda Dental Hygienist, Health Promotion Section, Aoba Ward Welfare and Health Center, Kanagawa, Japan. Discussed the results, reviewed and edited the manuscript.; e Mayuka Asaeda Researcher, Department of Oral Epidemiology, Graduate School of Biomedical and Health Sciences, Hiroshima University, Hiroshima, Japan. Discussed the results, reviewed and edited the manuscript.; f Yukoh Muraki Dentist, Dentistry and Oral Surgery, Japan Community Health Care Organization (JCHO) Tokuyama Central Hospital, Yamaguchi, Japan. Discussed the results, reviewed and edited the manuscript.; g Noriyasu Uchibori Dentist, Aichi Dental Association, Aichi, Japan. Discussed the results, reviewed and edited the manuscript.; h Mariko Naito Professor, Department of Oral Epidemiology, Graduate School of Biomedical and Health Sciences, Hiroshima University, Hiroshima, Japan. Conceived the study, administered surveys to the study population, and reviewed and edited the manuscript.

**Keywords:** cross-sectional study, history of systemic disease, older people, oral health, oral hygiene instruction

## Abstract

**Purpose:**

Inadequate oral hygiene and management affect oral health and are associated with systemic diseases. Although systemic disease history may relate to subjective oral status and oral health behaviours, these associations remain insufficiently examined. This study investigated the associations between systemic disease history, subjective oral status, and oral health behaviours among community-dwelling older adults.

**Methods and Materials**: We analysed data from 979 individuals aged 65–85 years, residing in Higashiura-cho, Aichi Prefecture, who participated in a 2018 group examination and completed a self-administered questionnaire. Multivariable logistic regression was used to evaluate associations between oral-related variables (‘concerns about teeth and mouth condition’, ‘use of interdental brushes and dental floss’, and ‘experience of oral hygiene instruction’) and histories of systemic diseases (cancer, myocardial infarction, stroke, hypertension, diabetes, liver disease, lung disease, mental illness, and bone disease).

**Results:**

The mean age was 73.0 ± 5.2 years, and 52.4% were female. No significant associations were observed between ‘concerns about teeth and mouth condition’, ‘use of interdental brushes and dental floss’, and systemic disease history. However, participants who had received oral hygiene instruction had higher odds of a history of hypertension (odds ratio: 1.4; 95% confidence interval: 1.0–2.0).

**Conclusion:**

Among community-dwelling older adults, receiving oral hygiene instruction was positively associated with a history of hypertension. As the data were self-reported, findings should be interpreted with caution. Dental hygienists should consider patients’ systemic health status, particularly hypertension, when providing oral hygiene instruction.

Common oral diseases, such as dental caries, periodontal disease, and oral cancer, affect almost half (45%) of the world’s population; 3.5 billion people live with untreated oral diseases.^5^ Poor oral health is linked to poor general health, reduced activities of daily living, and quality of life.^23^ Additionally, lower oral health-related quality of life and tooth loss have been associated with increased mortality.^3,14
^ The importance of appropriate self-care is widely recognised,^36^ and interventions are being developed to promote this practice.^11^ Effective self-care in oral hygiene management is essential for maintaining oral health.^8^


Various systemic diseases, including cardiovascular, respiratory, gastrointestinal, and metabolic diseases (e.g. diabetes), are associated with oral health.^6,9,33
^ Moreover, many previous studies have reported that periodontal disease affects systemic health.^10,17,35
^


Periodontal disease, a chronic inflammatory condition that affects periodontal tissues, is negatively correlated with oral health-related quality of life.^1^ Its development is influenced by the accumulation of dental plaque and tartar resulting from inadequate oral hygiene and by a dysbiosis of the oral microbiome and alterations in the host inflammatory response.^24^ Through these complex interactions, periodontal disease contributes to periodontal tissue destruction and has been linked to systemic inflammation and metabolic syndrome.^2,30
^ Consequently, maintaining proper oral hygiene and preventive oral healthcare is important for preventing the progression of periodontal disease and its systemic implications, particularly in individuals with chronic health conditions.

Oral health status is reflected in both clinical and self-assessment measures.^13^ Several factors associated with oral self-assessment, such as pain and difficulty in chewing,^29^ are also related to systemic health.^4,18
^ Subjective oral health status is typically assessed using symptoms such as pain and gingival swelling,^20,31
^ whereas subjective oral health behaviours are commonly measured using indicators such as tooth brushing frequency.^7^ Although subjective oral status and oral health behaviours are important for maintaining and improving oral health, the association between a history of systemic diseases^15,27
^ and these oral factors has not been fully clarified. Previous studies have mainly focused on specific disease outcomes, and comprehensive analyses incorporating both subjective oral status and behavioural factors in relation to systemic disease history, particularly among community-dwelling older adults, remain limited. Therefore, this cross-sectional study aimed to examine the association between a history of systemic diseases, subjective oral status, and oral health behaviours in community-dwelling older adults using data obtained from self-administered questionnaires without accompanying clinical oral examinations.

## Methods and Materials

### Study Design and Participants

The data analysed were obtained from the study titled ‘Verification of the support for independence and prevention of serious illness among the elderly through dental check-ups and follow-up, as well as the assessment of a comprehensive programme including maintenance of oral function, nutrition, and exercise’. This study was conducted in 2018 among individuals aged 65–85 years residing in Higashiura-cho, Aichi Prefecture, and data were collected at the end of June 2018. A self-administered questionnaire was enclosed with the information about the group examination sent to 10,016 participants, after excluding those requiring nursing care and support. The completed questionnaires were collected at the group examination venue. Of the 1,210 individuals who participated in the screening, 979 individuals without missing values were included in the analysis, and a cross-sectional study was conducted (Fig 1).

**Fig 1 Fig1:**
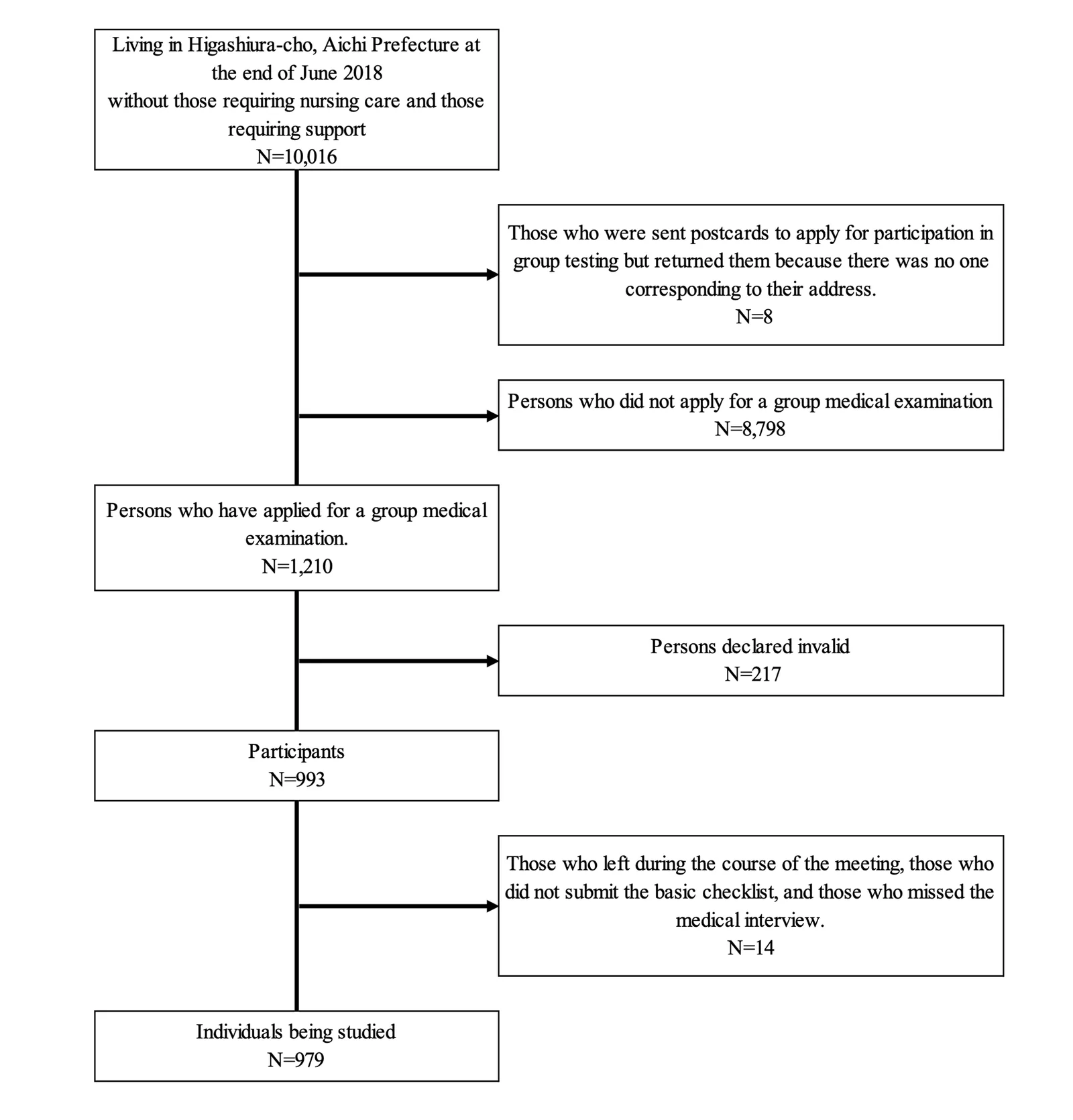
Flow diagram of study participants.

### Self-Administered Questionnaire

The self-administered questionnaire consisted entirely of dichotomous (‘yes/no’) items. For oral-health-related variables, participants were asked the following three questions: ‘Do you have concerns about the condition of your teeth and mouth?’ ‘Do you use interdental brushes or dental floss?’, and ‘Have you ever received oral hygiene instruction?’. These three questions were selected because they represent the key domains of oral health assessment: subjective oral health status (concerns about the teeth and mouth), oral health behaviours (use of interdental cleaning devices), and exposure to professional oral hygiene instructions. Regarding systemic disease history, participants were asked, ‘Have you ever received treatment or are you currently receiving treatment for any of the following diseases?’. The listed diseases included cancer, myocardial infarction, stroke, hypertension, diabetes, hepatitis, liver cirrhosis, lung diseases such as chronic obstructive pulmonary disease and pneumonia, mental illness, bone diseases such as osteoporosis, and an ‘other’ category with a free-response option. Only participants who completed all the relevant items without missing values were included in the analysis.

### Statistical Analysis

The chi-squared test was used to compare categorical variables, and either the t-test or Mann–Whitney U test was used to compare continuous variables, depending on the data distribution. Logistic regression analysis was performed with oral factors as outcome variables and medical history as explanatory variables. Model goodness of fit was evaluated using the Hosmer–Lemeshow test. Prior to the regression analyses, chi-squared tests were conducted to examine associations among systemic disease variables as an exploratory assessment of potential dependency between medical conditions.

Model 1 included sex and age as covariates, and Model 2 added the number of previous medical conditions (categorised as one, two, three, or more) to the variables included in Model 1. Model 3 was adjusted for smoking habits. Smoking history was categorised as ‘yes’ or ‘no’ based on responses to the questions: ‘Do you smoke cigarettes?’ and ‘Have you ever smoked cigarettes?’.

Statistical analyses were performed using IBM SPSS Statistics version 28 (IBM, Armonk, NY, USA). *p* values of < 0.05 were considered indicative of statistically significant differences. Participants were informed about the study, and written consent for research involvement was obtained. The study was approved by the relevant ethics committee (E2021-2704). This study was conducted in accordance with the Strengthening the Reporting of Observational Studies in Epidemiology (STROBE) statement.

## Results

### Participant Characteristics

The mean age ( ± standard deviation) of the participants was 73.0 ± 5.2 years, and women accounted for 52.4% of the sample (Table 1). Regarding medical history, 14.5% of the participants had cancer, and 41.5% had hypertension. Additionally, 70.9% and 80.4% of respondents reported using interdental brushes or dental floss and receiving oral hygiene instructions, respectively.

**Table 1 Table1:** Characteristics of the participants

Characteristics	Total (n = 979)	Male (n = 466)	Female (n = 513)	*p* value
Age, y ± SD	73.0 ± 5.2	73.0 ± 5.2	73.0 ± 5.3	0.298^‡^
BMI, kg/m^2^ ± SD	22.6 ± 3.0	23.0 ± 2.8	22.0 ± 3.1	< 0.001^§^
Disease				
Cancer, N (%)	142 (14.5)	85 (18.3)	57 (11.1)	0.002^||^
Myocardial infarction, N (%)	68 (7.0)	43 (9.3)	25 (4.9)	0.007^||^
Stroke, N (%)	27 (2.8)	17 (3.7)	10 (2.0)	0.105^||^
Hypertension, N (%)	405 (41.5)	220 (47.3)	185 (36.2)	< 0.001^||^
Diabetes, N (%)	122 (12.5)	78 (16.8)	44 (8.6)	< 0.001^||^
Liver disease*, N (%)	27 (2.8)	18 (3.9)	9 (1.8)	0.044^||^
Lung disease^†^, N (%)	55 (5.7)	29 (6.3)	26 (5.1)	0.431^||^
Mental illness, N (%)	14 (1.4)	4 (0.9)	10 (2.0)	0.152^||^
Osteoporosis, N (%)	104 (10.7)	9 (1.9)	95 (18.6)	< 0.001^||^
Have concerns about teeth or mouth, N (%)	464 (47.5)	202 (43.5)	262 (51.2)	0.016^||^
Use interdental brushes and floss, N (%)	693 (70.9)	300 (64.5)	393 (76.6)	< 0.001^||^
Have received oral hygiene instruction, N (%)	785 (80.4)	361 (77.6)	424 (83.0)	0.036^||^
* Hepatitis, liver cirrhosis ^†^ COPD, pneumonia ^‡^ Comparison of men and women using a Mann–Whitney U test. ^§^ Comparison of men and women using a t-test. ^||^ Comparison of men and women using a chi-squared test.				

### Distribution of Oral-Related Variables and Systemic Disease History

The bivariate associations among subjective oral status, oral health behaviours, and systemic disease history are shown in Table 2. No significant associations were observed between subjective oral status, oral health behaviours, and a history of systemic diseases in either men or women. However, participants with a history of osteoporosis showed a higher proportion reporting concerns about the condition of their teeth and mouth (*p* = 0.045) and the use of interdental brushes or dental floss (*p* = 0.019) than those without a history of osteoporosis.

**Table 2 Table2:** Association of subjective oral status and oral health behaviours with a history of systemic disease

Disease	Medical history	Total (n = 979)						Male (n = 466)						Female (n = 513)					
		Have concerns about the condition of teeth or mouth		Use interdental brushes and floss		Have received oral hygiene instruction		Have concerns about the condition of teeth or mouth		Use interdental brushes and floss		Have received oral hygiene instruction		Have concerns about the condition of teeth or mouth		Use interdental brushes and floss		Have received oral hygiene instruction	
		N (%)	*p* value*	N (%)	*p* value*	N (%)	*p* value*	N (%)	*p* value*	N (%)	*p* value*	N (%)	*p* value*	N (%)	*p* value*	N (%)	*p* value*	N (%)	*p* value*
Cancer	Yes	64 (45.1)	0.524	95 (66.9)	0.253	120 (84.5)	0.194	36 (42.4)	0.823	56 (65.9)	0.771	69 (81.2)	0.386	28 (49.1)	0.731	39 (68.4)	0.114	51 (89.5)	0.175
	No	400 (48.0)		598 (71.6)		655 (79.8)		166 (43.7)		244 (64.2)		292 (76.8)		234 (51.5)		354 (77.8)		373 (82.3)	
Myocardial infarction	Yes	26 (38.2)	0.117	50 (73.5)	0.621	56 (82.9)	0.688	13 (30.2)	0.069	27 (62.8)	0.800	32 (74.4)	0.600	13 (52.0)	0.925	23 (92.0)	0.062	24 (96.0)	0.075
	No	436 (48.1)		642 (70.7)		728 (80.4)		188 (44.7)		273 (64.8)		329 (78.1)		248 (51.0)		369 (75.8)		399 (82.3)	
Stroke	Yes	15 (55.6)	0.392	19 (70.4)	0.960	22 (81.5)	0.887	7 (41.2)	0.848	13 (76.5)	0.294	14 (82.4)	0.634	8 (80.0)	0.065	6 (60.0)	0.212	8 (80.0)	0.803
	No	448 (47.2)		673 (70.8)		762 (80.4)		195 (43.5)		287 (64.1)		347 (77.5)		253 (50.5)		386 (76.9)		415 (83.0)	
Hypertension	Yes	203 (50.2)	0.147	282 (69.6)	0.499	333 (82.4)	0.178	107 (48.6)	0.032	133 (60.5)	0.083	174 (79.1)	0.475	96 (52.2)	0.735	149 (80.5)	0.106	159 (86.4)	0.114
	No	260 (45.5)		409 (71.6)		450 (78.9)		95 (38.8)		167 (68.2)		187 (76.3)		165 (50.6)		242 (74.2)		263 (80.9)	
Diabetes	Yes	58 (47.5)	0.981	79 (64.8)	0.115	95 (77.9)	0.450	33 (42.3)	0.825	45 (57.7)	0.167	62 (79.5)	0.667	25 (56.8)	0.425	34 (77.3)	0.907	33 (75.0)	0.143
	No	405 (47.4)		613 (71.7)		689 (80.8)		169 (43.7)		255 (65.9)		299 (77.3)		236 (50.5)		358 (76.5)		390 (83.7)	
Liver disease^†^	Yes	13 (48.1)	0.940	22 (81.5)	0.217	23 (85.2)	0.526	7 (38.9)	0.691	14 (77.8)	0.230	15 (83.3)	0.554	6 (66.7)	0.345	8 (88.9)	0.378	8 (88.9)	0.632
	No	450 (47.4)		670 (70.5)		761 (80.3)		195 (43.6)		286 (64.0)		346 (77.4)		255 (50.8)		384 (76.3)		415 (82.8)	
Lung disease^‡^	Yes	33 (60.0)	0.054	40 (72.7)	0.766	47 (85.5)	0.339	17 (58.6)	0.090	23 (79.3)	0.094	25 (86.2)	0.267	16 (61.5)	0.269	17 (65.4)	0.173	22 (84.6)	0.805
	No	427 (46.7)		649 (70.9)		733 (80.2)		184 (42.5)		277 (64.0)		335 (77.4)		243 (50.4)		372 (77.0)		398 (82.7)	
Mental illness	Yes	10 (71.4)	0.071	10 (71.4)	0.965	11 (78.6)	0.863	2 (50.0)	0.793	3 (75.0)	0.664	2 (50.0)	0.184	8 (80.0)	0.065	7 (70.0)	0.621	9 (90.0)	0.549
	No	453 (47.1)		682 (70.9)		772 (80.4)		200 (43.5)		297 (64.6)		358 (77.8)		253 (50.5)		385 (76.7)		414 (82.8)	
Osteoporosis	Yes	59 (56.7)	0.045	84 (80.8)	0.019	87 (83.7)	0.401	5 (55.6)	0.600	5 (55.6)	0.564	5 (55.6)	0.105	54 (56.8)	0.205	79 (83.2)	0.091	82 (86.3)	0.354
	No	403 (46.3)		607 (69.7)		697 (80.2)		197 (43.3)		295 (64.8)		356 (78.2)		206 (49.6)		312 (75.0)		341 (82.4)	
* Chi-squared test. ^†^ Hepatitis, liver cirrhosis ^‡^ COPD, pneumonia																			

### Associations Between Systemic Disease History and Oral-Related Factors

Results of the multivariable logistic regression analyses are presented in Table 3.

**Table 3 Table3:** Association of subjective oral status and oral health behaviours with a history of systemic disease

Disease	Model 1						Model 2						Model 3					
	Have concerns about the condition of teeth or mouth		Use interdental brushes and floss		Have received oral hygiene instruction		Have concerns about the condition of teeth or mouth		Use interdental brushes and floss		Have received oral hygiene instruction		Have concerns about the condition of teeth or mouth		Use interdental brushes and floss		Have received oral hygiene instruction	
	OR (95% CI)^‡^	*p* value	OR (95% CI)^‡^	*p* value	OR (95% CI)^‡^	*p* value	OR (95% CI)^§^	*p* value	OR (95% CI)^§^	*p* value	OR (95% CI)^§^	*p* value	OR (95% CI)^‖^	*p* value	OR (95% CI)^‖^	*p* value	OR (95% CI)^‖^	*p* value
Cancer	0.9 (0.7–1.3)	0.731	0.9 (0.6–1.3)	0.463	1.5 (0.9–2.4)	0.125	0.7 (0.5–1.1)	0.160	0.8 (0.5–1.2)	0.222	1.3 (0.7–2.2)	0.405	0.8 (0.5–1.2)	0.242	0.7 (0.5–1.2)	0.201	1.2 (0.7–2.1)	0.527
Myocardial infarction	0.8 (0.5–1.3)	0.284	1.3 (0.7–2.2)	0.426	1.3 (0.7–2.4)	0.472	0.6 (0.3–1.1)	0.092	1.4 (0.7–2.7)	0.298	1.1 (0.5–2.3)	0.797	0.6 (0.3–1.1)	0.081	1.4 (0.8–2.7)	0.282	1.1 (0.5–2.2)	0.841
Stroke	1.5 (0.7–3.3)	0.300	1.1 (0.5–2.5)	0.882	1.1 (0.4–3.0)	0.786	1.4 (0.6–3.3)	0.403	1.2 (0.5–2.9)	0.741	1.1 (0.4–3.5)	0.809	1.5 (0.7–3.5)	0.319	1.2 (0.5–3.1)	0.684	1.1 (0.4–3.4)	0.859
Hypertension	1.3 (1.0–1.7)	0.031	0.96 (0.7–1.3)	0.773	1.3 (0.97–1.9)	0.079	1.3 (0.99–1.7)	0.056	0.98 (0.7–1.3)	0.899	1.4 (1.0–2.0)	0.044	1.3 (0.96–1.6)	0.094	0.95 (0.7–1.3)	0.737	1.4 (1.0–2.0)	0.038
Diabetes	1.1 (0.8–1.6)	0.605	0.8 (0.5–1.2)	0.269	0.9 (0.6–1.4)	0.681	0.98 (0.6–1.5)	0.912	0.7 (0.4–1.1)	0.136	0.7 (0.4–1.2)	0.190	0.95 (0.6–1.5)	0.812	0.7 (0.4–1.1)	0.142	0.7 (0.4–1.2)	0.163
Liver disease*	1.1 (0.5–2.4)	0.768	2.0 (0.8–5.6)	0.148	1.5 (0.5–4.5)	0.438	0.8 (0.4–1.9)	0.679	2.7 (0.9–8.2)	0.086	1.6 (0.5–5.9)	0.442	0.8 (0.3–1.8)	0.509	2.6 (0.8–7.9)	0.102	2.5 (0.6–11.2)	0.223
Lung disease^†^	1.8 (1.0–3.2)	0.032	1.1 (0.6–2.1)	0.715	1.5 (0.7–3.3)	0.288	1.6 (0.9–3.0)	0.108	1.1 (0.5–2.0)	0.852	1.5 (0.6–3.5)	0.367	1.5 (0.8–2.8)	0.163	1.0 (0.5–2.0)	0.914	1.8 (0.7–4.4)	0.230
Mental illness	2.6 (0.8–8.4)	0.110	0.9 (0.3–3.0)	0.888	0.8 (0.2–3.0)	0.767	2.3 (0.7–7.6)	0.174	0.9 (0.3–2.9)	0.833	0.6 (0.2–2.4)	0.508	2.5 (0.7–8.2)	0.142	0.9 (0.3–3.0)	0.880	0.6 (0.2–2.3)	0.478
Osteoporosis	1.4 (0.9–2.2)	0.097	1.4 (0.8–2.4)	0.182	1.1 (0.6–2.0)	0.705	1.3 (0.8–2.0)	0.292	1.5 (0.9–2.8)	0.153	0.9 (0.5–1.6)	0.669	1.3 (0.8–2.1)	0.266	1.6 (0.9–2.8)	0.136	0.9 (0.5–1.6)	0.633
* Hepatitis, liver cirrhosis ^†^ COPD, pneumonia ^‡^ Binomial logistic regression analysis; Model 1 was adjusted for age and sex. ^§^ Binomial logistic regression analysis; Model 2 was adjusted for all variables in Model 1 and number of previous systemic diseases (one, two, three, or more). ^||^ Binomial logistic regression analysis; Model 3 was adjusted for all variables in Model 2 and smoking status.																		

No significant associations were observed between the use of interdental brushes or dental floss and systemic disease history across all models.

Participants who reported concerns about the condition of their teeth and mouth showed lower odds of having a history of myocardial infarction compared with those who did not report such concerns (odds ratio [OR]: 0.6; 95% confidence interval [CI]: 0.3–1.1) in Models 2 and 3; however, this association was not statistically significant.

In contrast, participants who had received oral hygiene instruction had higher odds of having a history of hypertension compared with those who had not received such instruction (OR: 1.4; 95% CI: 1.0–2.0) in Models 2 and 3.

The goodness of fit for all multivariable logistic regression models was acceptable, with *p* values from the Hosmer–Lemeshow test > 0.05. In exploratory chi-squared analyses, no statistically significant associations were observed among the systemic disease variables.

## Discussion

The present results indicate that those who received oral hygiene instruction were more likely to have a history of hypertension than those who did not. These findings suggest that dental care may be an important platform for delivering preventive and lifestyle-related interventions to patients with systemic conditions. Miyawaki et al^26^ reported that hypertension has the highest prevalence (10.9%) among Japanese adult dental patients. Additionally, the 2018 National Health and Nutrition Survey in Japan reported a hypertension prevalence of 66.8% among adults aged 65–74 years.^28^ In the present study, more than 40% of the participants had a history of hypertension, indicating a higher prevalence of hypertension than other chronic conditions. Incorporating information on systemic diseases into oral health education may facilitate comprehensive and individualised care for older adults.

However, this association cannot be explained by prevalence alone. In dental practice, measuring blood pressure before treatment is recommended as part of patient safety management,^16^ which may increase the likelihood of identifying patients with hypertension during dental visits. Furthermore, these individuals are prone to deterioration of their oral health due to the side effects of therapeutic drugs,^16^ thereby increasing opportunities for dental professionals to provide targeted oral hygiene instruction and continuous preventive care. In Japan, a dental hygienist, under the direction of the attending dentist, provides direct practical guidance to patients for at least 15 min and provides written information on the content of the guidance.^25^ The significant relationship observed between receiving oral hygiene instructions and having a history of hypertension may be attributed to this factor.

From a statistical perspective, the association between a history of hypertension and receipt of oral hygiene instruction showed a tendency toward significance in Model 1 (OR: 1.3; 95% CI: 0.97–1.9; *p* = 0.079), which was adjusted only for age and sex, and became statistically significant after further adjustment in Models 2 and 3. Lehnert et al reported that older adults with multiple chronic conditions use medical services more frequently. Thus, connecting them to regular check-ups and health guidance may lead to increased interest in and adoption of health behaviours.^21^ Additionally, smoking is a major risk factor that adversely affects both systemic and oral health,^34^ and nonsmokers tend to be more health-conscious.^12^ In particular, patients with multiple conditions may have increased opportunities to receive guidance on the importance of oral care as part of comprehensive lifestyle interventions.^21^ Therefore, the observed association between hypertension and oral health behaviours may reflect the influence of these shared confounding factors rather than the direct effects of hypertension itself. These results are consistent with those of a previous study^19^ showing that oral health behaviours are more closely related to patients’ overall health status and lifestyle patterns than to associations with single diseases.

Although the link between diabetes and periodontal disease is widely recognised,^22^ the present study found no significant association between diabetes and subjective oral status or oral health behaviours. In this study, participants were not certified as requiring support or long-term care under Japan’s long-term care insurance system and were able to attend the health examination venue independently. Previous studies have shown that well-managed diabetes has a smaller impact on periodontal health than poorly controlled diabetes.^32^ Additionally, periodontal disease is often asymptomatic in its early stages, with noticeable symptoms, such as pain or swelling, typically occurring only in moderate to severe cases. Future studies should include more detailed information on the diabetes management status, such as glycemic control levels and objective measures of periodontal disease severity, to better understand the relationship between diabetes and oral health behaviours.

The strength of the present study is that it was based on a relatively large sample size and examined the association between a history of systemic disease and oral-related factors. However, this study had several limitations. First, its cross-sectional design precluded causal inferences. Second, the systemic disease history was self-reported and lacked detailed information on disease severity, treatment status, and duration. Third, the study population was limited to a single region, which may have affected generalizability. Fourth, the subjective oral status assessed may not accurately reflect specific oral diseases, and oral health behaviours, such as the use of interdental cleaning devices, may not necessarily indicate their effectiveness.

An additional limitation of this study is the reliance on self-administered questionnaires without clinical verification. Self-reported oral status, oral health behaviours, and systemic disease history may not accurately reflect actual clinical conditions, behavioural effectiveness, or disease severity, thereby limiting the precision of the findings. Moreover, differences in sample sizes across disease groups, particularly the larger number of participants with hypertension, may have influenced the likelihood of detecting significant associations. Smoking status was assessed dichotomously (yes/no), preventing evaluation of dose–response relationships. Future studies should employ more detailed smoking measures, such as never/former/current smoking status, smoking duration, and pack-years, to better assess the impact of tobacco exposure. Finally, although we adjusted for age, sex, and smoking status, other factors such as functional status and frailty were not included, which may have influenced the observed associations. These findings are consistent with recent initiatives by the European Federation of Periodontology (EFP), the American Academy of Periodontology (AAP),^17^ and the World Health Organization (WHO),^37^ which advocate greater integration of oral and general healthcare and emphasise the importance of oral health in chronic disease prevention and management. A longitudinal study of oral status, health behaviours, and the risk of developing systemic diseases is warranted to validate these associations.

## Conclusions

This study evaluated the associations between subjective oral status, oral health behaviours, and a history of systemic diseases among community-dwelling older adults using self-administered questionnaire data. Receipt of oral hygiene instruction was significantly associated with a history of hypertension, highlighting the potential role of dental professionals in integrated oral–systemic healthcare. These findings further highlight that dental settings may provide valuable opportunities to address broader health concerns in ageing populations with a high burden of chronic diseases. However, because all variables were assessed through self-report without clinical verification, further studies incorporating objective clinical assessments are warranted to confirm and extend these findings.

### Conflicts of interest

The authors declare no conflict of interest.

### Funding

This work was supported by a Health and Labour Sciences Research Grant from the Ministry of Health, Labour, and Welfare, Japan (Project for Promotion of Health Services for the Elderly, 2018).

## References
